# In vitro effect of zirconia type on shear bond strength to feldspathic porcelain and wear of the opposing teeth

**DOI:** 10.34172/joddd.40755

**Published:** 2024-06-24

**Authors:** Amirhossein Rafiei, Vahid Fakhrzadeh, Elnaz moslehifard, Ghazal Ranjbar

**Affiliations:** ^1^Department of Prosthodontics, Faculty of Dentistry, Tabriz University of Medical Sciences, Tabriz, Iran; ^2^Student Research Committee, Faculty of Dentistry, Shahid Beheshti University of Medical Sciences, Tehran, Iran

**Keywords:** Dental porcelain, Shear bond strength, Tooth wear, Zirconium

## Abstract

**Background.:**

Multilayer zirconia has more optical and aesthetic features than regular zirconia. Therefore, its mechanical properties should be compared with monochromatic zirconia. Among the mechanical characteristics that can be checked are the wear of the opposite tooth and the bond to the porcelain. This study assessed the effect of zirconia type (multilayer versus monochromatic) on the shear bond strength (SBS) to feldspathic porcelain and the wear of the opposing teeth.

**Methods.:**

The present in vitro study was conducted in two phases. In the first phase, 15 multilayer and 15 monochromatic zirconia blocks measuring 10×5×5 mm were designed, milled, sintered, veneered with porcelain, and underwent thermocycling. Their SBS was then measured in a universal testing machine. In the second phase, 15 multilayer and 15 monochromatic zirconia blocks were placed in a chewing simulator, and 30 sound premolars served as antagonistic teeth. The magnitude of wear of the buccal cusp of premolars was quantified from a 4-mm reference point after 100000 cycles. Data were analyzed by independent *t* test (α=0.05).

**Results.:**

The mean SBS of monochromatic zirconia to porcelain (24.49±3.58 MP) was slightly higher than that of multilayer zirconia (22.98±2.98 MP), but the difference was not significant (*P*>0.05). The mean wear of the opposing teeth was also slightly higher in the monochromatic group (284.1±66.53 µm) than in the multilayer group (263.2±58.69 µm), but this difference was not significant either (*P*>0.05).

**Conclusion.:**

Monochromatic and multilayer zirconia showed comparable SBS to feldspathic porcelain and caused comparable wear of the opposing teeth in vitro. Thus, multilayer zirconia may serve as an alternative to monochromatic zirconia.

## Introduction

 In recent years, digital dentistry has profoundly improved the quality and efficacy of many dental treatments.^1^ Advances in dental materials science coupled with the advent of digital dentistry have contributed to the routine selection of tooth-colored all-ceramic restorations in dental treatment planning.^2^ Zirconium is a highly popular dental material in digital dentistry. It has favorable mechanical properties such as high fracture resistance, optimal chemical stability, and acceptable biocompatibility.^3^ Nonetheless, aesthetics is a major concern when using zirconium since it does not allow optimal simulation of color and details of natural teeth.^4^ The conventional zirconia is monochromatic and has low translucency, which limits its application in the esthetic zone.^5^ To overcome such shortcomings, feldspathic zirconia veneering has been suggested, which is routinely performed in dental practice. However, chipping and delamination of porcelain are a common occurrence that is among the main causes of failure of such treatments^6^ and occur due to incompatibility of the thermal properties of zirconia and porcelain.^7^

 To overcome the poor aesthetic appearance of monochromatic zirconia and the problems associated with porcelain veneering of the zirconia framework, multilayer zirconia was introduced in recent years, which can have variable color properties from cervical to incisal areas.^8^ The translucency of zirconia may be improved by decreasing its Al_2_O_3_ content during sintering, increasing the sintering temperature, and changing the cubic content (controlled by the amount of yttria and sintering temperature). Higher yttria content and higher sintering temperature increase the cubic content and result in higher translucency; however, the mechanical properties of zirconia would decrease.^9^ In multilayer zirconia, the highest opacity and chroma are at the cervical third, and translucency increases toward the incisal third.

 Although multilayer zirconia has been proposed as an alternative to porcelain veneering of monochromatic zirconia, porcelain veneering of multilayer zirconia can also be performed for higher aesthetics. Porcelain chipping is among the most common causes of failure of zirconia bridges due to the absence of a strong bond between the porcelain and zirconia.^10,11^ The prevalence of porcelain‒zirconia debonding ranges from 6% to 15% at 3‒5-year follow-ups, which is higher than in crowns with a metal core.^12^ Unlike metal, the mechanism of bonding of ceramic to zirconia has not been well elucidated. Nonetheless, the wettability of zirconia and chemical and micromechanical bonding play a role in this regard.

 Wear of the opposing teeth is another crucial factor to consider in prosthetic restorations. The abrasive activity of restorative materials on the opposing natural dentition has never been eliminated despite the continuous advancements in these materials and continues to be a clinical challenge.^13^ Zirconia can cause high wear of the opposing teeth due to its high hardness and strength, which is a concern in clinical practice.^14^ Enamel wear can occur for several reasons and leads to loss of vertical height of occlusion, poor aesthetics, tooth hypersensitivity, decreased efficiency of mastication, and temporomandibular problems.^15^ The mechanism of wear may include erosion, abrasion, or attrition; commonly, wear occurs due to a combination of the phenomena mentioned above.^16^ Zirconia has higher hardness (13 Gpa) than ceramic and enamel (3.14‒3.72 Gpa) and can cause significant wear of the opposing teeth. However, it has been reported that the wear of the tooth against zirconia is lower than that against feldspathic porcelain, which can be attributed to the high fracture resistance of zirconia and the preservation of the flattened surface.^17^

 The authors’ literature search yielded no study comparing multilayer and monochromatic zirconia regarding their shear bond strength (SBS) to porcelain and wear of the opposing teeth. Also, a previous study reported lower tooth wear against monolithic zirconia compared with porcelain veneered zirconia.^5^ Thus, further investigations are warranted on tooth wear caused by multilayer and monochromatic zirconia. Considering all the above, this study aimed to assess the effect of zirconia type (multilayer versus monochromatic) on SBS to feldspathic porcelain and the wear of the opposing teeth. The first null hypothesis of the study was that no significant difference would be found in SBS of multilayer and monochromatic zirconia to feldspathic porcelain. The second null hypothesis of the study was that no significant difference would be found in the wear of the opposing teeth between the two zirconia groups.

## Methods

 This in vitro study was conducted in two phases. The SBS of multilayer and monochromatic zirconia to feldspathic porcelain was measured in the first phase. In the second phase, the wear of the opposing teeth caused by multilayer and monochromatic zirconia was assessed.

###  Sample size 

 The minimum sample size was calculated at n = 15 in each group according to a previous study by Choi et al,^18^ assuming a mean difference of 5.5 units in SBS, a standard deviation of 5.23, a type one error of 0.05, and a study power of 80%.

###  Specimen preparation 

**Figure 1:**
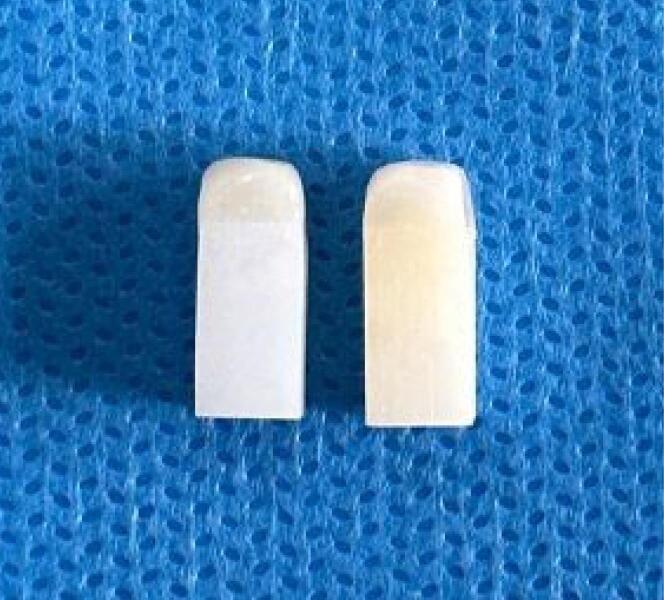
Porcelain veneered zirconia blocks: (right) multilayer; (left) monochromatic

 Exocad software was used to design 15 multilayer zirconia (Pritidenta Multilayer HT, Pritidenta® GmbH, Germany) and 15 monochromatic zirconia (Pritidenta WO, Pritidenta®GmbH, Germany) blocks measuring 10 × 5 × 5 mm.^17^ The file was sent to a milling machine, and after completing the milling process, sintering was performed as instructed by the manufacturer. The surface to be veneered with porcelain was then sandblasted with 110-µm Al_2_O_3_ particles with 2.5-bar pressure (Koosha Fan Pars, Tehran, Iran).^18^ The blocks were then cleaned and dried, and the veneering porcelain (CZR Ceramic porcelain; Kuraray Noritake Inc., Japan) was applied on the sandblasted zirconia surface (5 × 5 mm) with 3-mm thickness (Figure 1). Considering the volumetric changes of porcelain after firing, porcelain had to be added in several steps because the external angle of the porcelain‒zirconia interface had to be ≤ 180º to prevent slipping of the blade of the universal testing machine. The specimens were then placed in a furnace for porcelain firing.

###  Thermocycling

 All the specimens underwent thermocycling (Dorsa, Iran) for 10 000 cycles at 5‒60 °C with a dwell time of 30 seconds and a transfer time of 10 seconds.

###  SBS test 

 Each block was then mounted in acrylic resin (Acropars 200; Marlik, Tehran, Iran). The SBS of zirconia to porcelain was measured in a universal testing machine (STM20; Santam, Iran).^18^ Load was applied to the zirconia‒porcelain interface by a chisel at a speed of 1 mm/minute. The exact location of the interface was first marked by a marker. The load was applied until the porcelain was debonded from zirconia, and the magnitude of force causing debonding was recorded. The SBS was measured by dividing the load at debonding by the surface area.

###  Tooth wear

**Figure 2:**
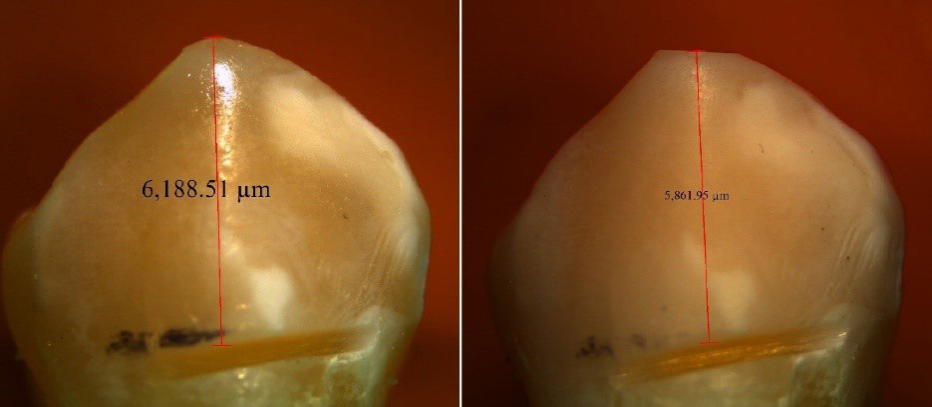
Quantifying wear under a stereomicroscope at × 10 magnification by measuring the distance between the created groove and the buccal cusp tip; (Left) before the wear test; (Right) after the wear test

 A total of 15 multilayer and 15 monochromatic zirconia blocks measuring 10 × 5 × 5 mm were milled as presintered. The blocks were sintered as instructed by the manufacturer and polished with polishing discs. Thirty sound premolars with no cracks or caries, extracted for reasons unrelated to this study (such as orthodontic treatment or periodontal disease), were used as antagonists.^4^ The teeth were sterilized and cleaned with pumice paste. Next, the teeth and zirconia blocks were individually mounted in cylindrical auto-polymerizing acrylic resin (Acropars 200, Marlik, Tehran, Iran). The teeth were mounted such that their buccal cusp was positioned at a level higher than their lingual cusp (to undergo wear). The specimens were then placed in a chewing simulator (Nemo Mechatronic, Nemo Fanavaran Pars, Mashhad, Iran).^19^ This device simulated the vertical and horizontal movements of the masticatory function. The specimens and the teeth were immersed in water during load cycling to better simulate the oral environment. The teeth applied a 50-N vertical load (5 kg) to the block surface and then made a 1-mm sliding movement. This process was repeated 100 000 times. The wear of the buccal cusp was measured under a stereomicroscope (LEICA EZ4 D, Mel Sobel Microscopes Ltd., USA) at × 10 magnification. Prior to testing, a groove was created at a 4-mm distance from the buccal cusp tip, and the distance between the superior border of this groove and the cusp tip was measured under a stereomicroscope (Figure 2A). The same measurement was made after the wear test (Figure 2B), and the difference in values was calculated to determine the magnitude of wear. To maximize accuracy, each measurement was repeated four times.

###  Statistical analysis 

 The normal distribution of data was evaluated by the Kolmogorov-Smirnov test, which showed a normal distribution of SBS and wear data (*P* > 0.05). F-test was applied to analyze the homogeneity of variances, which showed that the assumption of variance homogeneity was met for both the SBS and wear data (*P* > 0.05). Thus, the SBS of the two types of zirconia and wear of the opposing teeth were compared by independent t-test. All the statistical analyses were performed using SPSS 20 (SPSS Inc., IL, USA) at an 0.05 level of significance.

## Results

###  SBS

 The comparison of SBS between monochromatic and multilayer zirconia revealed thaton average ( ± SD), monochromatic zirconia showed an SBS of 24.49 ± 3.58 MP compared with 22.98 ± 2.98 MP for multilayer zirconia. The mean SBS of monochromatic zirconia was slightly higher than that of multilayer zirconia, but the difference was not significant (*P* = 0.219) (Table 1).

**Table 1:** Mean SBS (MPa) of zirconia to porcelain and wear of the opposing teeth in the two groups of monochromatic and multilayer zirconia

**Variable**	**Monochromatic zirconia**		**Multilayer zirconia**		***P*** **value**^a^
	**Mean**	**SD**	**Mean**	**SD**	
SBS	24.49	3.58	22.98	2.98	0.219
Wear	284.1	66.53	263.2	58.69	0.370

SD, Standard deviation; SBS, Shear bond strength. ^a^ Independent *t *test.

###  Wear

 The mean wear of tooth samples ( ± SD) for monochromatic zirconia and multilayer zirconia was 284.1 ± 66.53 and 263.2 ± 58.69 µm. The mean wear of the opposing teeth was slightly higher by the monochromatic zirconia than by the multilayer zirconia, but the difference was not significant (*P* = 0.370) (Table 1).

## Discussion

 This study assessed the effect of zirconia type (multilayer versus monochromatic) on SBS to feldspathic porcelain and the wear of the opposing teeth. The mean SBS of monochromatic zirconia to porcelain was slightly higher than that of multilayer zirconia, but the difference was not significant (*P* > 0.05). The mean wear of the opposing teeth was also slightly higher in the monochromatic group than in the multilayer group, but this difference was not statistically significant either (*P* > 0.05). The first null hypothesis of the study was that no significant difference would be found in the SBS of multilayer and monochromatic zirconia to feldspathic porcelain, which was confirmed in this article.

 By searching the literature, no comparisons were found between the wear and SBSs of multilayer zirconia compared to monochromatic zirconia. As a result, the present study was designed and implemented to investigate and compare the wear and SBS.

 The SBS test was used in the present study since it has the highest reliability among different bond strength tests because the load is applied to the interface of two materials.^20^ Also, porcelain was applied by the layering technique since Abbasi et al^21^ and Teng et al^22^ showed that pressing and layering techniques had no significant difference concerning bond strength. The results showed that the mean SBS of monochromatic zirconia to porcelain was slightly higher than that of multilayer zirconia, but the difference was not significant (24.49 ± 3.58 versus 22.98 ± 2.98 MPa). Thus, the first null hypothesis of the study was accepted. This result is consistent with the findings of Choi et al,^18^ who reported that the bond strength of zirconia to porcelain was 25.43 ± 3.12 MPa. Also, Sreekala et al^23^ reported the SBS of zirconia to porcelain at 26.20 ± 1.20 MPa after aging. The difference between the reported values in their study and the present investigation can be attributed to the difference in the aging process adopted in the two studies. The results have been contradictory concerning the bond strength of zirconia to porcelain. Nishigori et al^24^ evaluated the effect of surface treatments on SBS of zirconia to porcelain and reported a bond strength of 34.1 ± 10 MPa after sandblasting with Al_2_O_3_ particles, which was higher than the values obtained in the two groups in the present study. This difference may be due to differences in the size of particles and sandblasting pressure, as well as in the dimensions of specimens. Nonetheless, they showed that sandblasting had no significant effect on the SBS of zirconia to porcelain, which is consistent with the results of Zandinejad et al^25^ and Abbasi et al.^21^

 The type of zirconia can also influence the efficacy of surface treatments. Aboushelib et al.^26^ indicated that sandblasting increased the surface roughness and SBS of white zirconia while it decreased the SBS of yellow zirconia. Different types of zirconia frameworks have different surface and material mass structural properties. Variations in particle size, shape, composition, density, and hardness are responsible for different effects of surface treatments on the final structure of zirconia.

 Thermocycling was also performed in the present study before SBS testing to better simulate the clinical setting. Ramos et al^3^ measured the bond strength of three types of zirconia to porcelain and showed that thermocycling did not significantly affect the results. However, Zandinejad et al^25^ assessed the SBS of zirconia fabricated by the computer-aided design/computer-aided manufacturing technology and stereolithography to porcelain with and without surface treatment and thermocycling. They reported that thermocycling significantly decreased the SBS in both groups.

 The second null hypothesis of the present study was that no significant difference would be found in the wear of the opposing teeth between the two zirconia groups. Assessment of the wear of the opposing teeth in the present study revealed that the mean wear of the opposing teeth was slightly higher in the monochromatic group than in the multilayer group, but this difference was not statistically significant (284.1 ± 66.53 versus 263.2 ± 58.69 μm). Thus, the second null hypothesis of the study was accepted. The wear values obtained in the present study were close to those reported by Vardhaman et al.^27^ However, the difference in wear caused by multilayer and 3Y-TZP zirconia in their study was statistically significant. The enamel layer of multilayer zirconia in their study had the highest cubic content and largest grain size. Two transitional layers were below the enamel layer and over the dentin layer. The 3Y-TZP zirconia had the largest grain size and the highest cubic content. They reported higher volume loss and greater wear depth in multilayer zirconia compared with 3Y-TZP. The wear pattern in multilayer zirconia was reported to be more heterogenous than that in 3Y-TZP, mainly due to the formation of extensive sub-surface cracks in multilayer zirconia, eventually resulting in local delamination of material and leading to further volume loss and greater depth of wear.^27^

 In the present study, the zirconia blocks were polished before testing. Chong et al^17^ reported that polishing and repolishing of zirconia surface after occlusal adjustment decreased the wear of antagonistic enamel, while unpolished zirconia caused wear comparable to that caused by enamel in the antagonistic enamel. The same results were reported by Gundugollo et al.,^4^ Steiner et al.,^28^ and Ghaffari et al,^29^ indicating that polished monolithic zirconia caused minimal wear in the antagonistic teeth. They also reported significantly lower wear caused by monolithic zirconia than layered zirconia. Stawarczyk et al^30^ evaluated the wear of antagonistic teeth by different materials and concluded that polished monolithic zirconia caused minimal wear among the tested materials. The specimens were placed in a chewing simulator for 50 000 cycles in the present study. Vardhaman et al^29^ applied 10 000 to 500 000 cycles, and Janyavula et al^31^ applied 200 000 and 400 000 cycles and reported that increasing the chewing cycles caused a significant difference in the wear of the opposing teeth.

 In vitro design was a limitation of this study, which limits the generalizability of the results to the clinical setting.

 Further research is indicated to compare multilayer zirconia with other ceramics such as lithium disilicate. To evaluate the wear, the amount of reduction in the height of the tooth was calculated. This measurement can be done as an evaluation of the contact surface.

## Conclusion

 Monochromatic and multilayer zirconia showed comparable SBS to feldspathic porcelain and caused comparable wear of the opposing teeth under in vitro conditions. Thus, multilayer zirconia may serve as an alternative to monochromatic zirconia.

## Acknowledgments

 This article was written based on a dataset from an MSc thesis entitled “In vitro evaluation of the effect of zirconia type, a marginal and internal fit, porcelain bond strength and opposing tooth wear in 3-unit fixed partial denture” registered at Tabriz University of Medical Sciences Faculty of Dentistry (reference number 70189). The thesis was supported by the Vice Chancellor for Research at Tabriz University of Medical Sciences.

## Competing Interests

 The authors declare no conflicts of interest.

## Ethical Approval

 The study protocol was approved by the Ethics Committee of Tabriz University of Medical Sciences (IR.TBZMED.VCR.REC.1401.413).

## Funding

 Tabriz University of Medical Sciences paid the costs of this study.

## References

[1] Suzuki S, Katsuta Y, Ueda K, Watanabe F (2020). Marginal and internal fit of three-unit zirconia fixed dental prostheses: effects of prosthesis design, cement space, and zirconia type. J Prosthodont Res.

[2] Song KH, Im YW, Lee JH, Lee J, Lee HH (2018). Evaluation of mold-enclosed shear bond strength between zirconia core and porcelain veneer. Dent Mater J.

[3] Ramos CM, Cesar PF, Lia Mondelli RF, Tabata AS, de Souza Santos J, Sanches Borges AF (2014). Bond strength and Raman analysis of the zirconia-feldspathic porcelain interface. J Prosthet Dent.

[4] Gundugollu Y, Yalavarthy RS, Krishna MH, Kalluri S, Pydi SK, Tedlapu SK (2018). Comparison of the effect of monolithic and layered zirconia on natural teeth wear: an in vitro study. J Indian Prosthodont Soc.

[5] Yotsuya M, Takuma Y, Sato T, Yasuda H, Sinya A, Sase T (2014). Evaluation of fitness of anterior zirconia ceramic bridge frame fabricated from intraoral digital impression. Shikwa Gakuho.

[6] Schley JS, Heussen N, Reich S, Fischer J, Haselhuhn K, Wolfart S (2010). Survival probability of zirconia-based fixed dental prostheses up to 5 yr: a systematic review of the literature. Eur J Oral Sci.

[7] Esquivel-Upshaw JF, Kim MJ, Hsu SM, Abdulhameed N, Jenkins R, Neal D (2018). Randomized clinical study of wear of enamel antagonists against polished monolithic zirconia crowns. J Dent.

[8] Kolakarnprasert N, Kaizer MR, Kim DK, Zhang Y (2019). New multi-layered zirconias: composition, microstructure and translucency. Dent Mater.

[9] Zhang Y (2014). Making yttria-stabilized tetragonal zirconia translucent. Dent Mater.

[10] Sailer I, Pjetursson BE, Zwahlen M, Hämmerle CH (2007). A systematic review of the survival and complication rates of all-ceramic and metal-ceramic reconstructions after an observation period of at least 3 years Part II: fixed dental prostheses. Clin Oral Implants Res.

[11] Sailer I, Fehér A, Filser F, Lüthy H, Gauckler LJ, Schärer P (2006). Prospective clinical study of zirconia posterior fixed partial dentures: 3-year follow-up. Quintessence Int.

[12] Augstin-Panadero R, Fons-Font A, Roman-Rodriguez JL, Granell-Ruiz M, del Rio-Highsmith J, Sola-Ruiz MF (2012). Zirconia versus metal: a preliminary comparative analysis of ceramic veneer behavior. Int J Prosthodont.

[13] Cherian J, Jayakumar R, James J, Thomas V, Sramadathil S, Kattachirakunnel Sasi A (2023). A comparative evaluation of enamel wear against different surface finished ceramics: an in vitro study. Cureus.

[14] Stober T, Bermejo JL, Rammelsberg P, Schmitter M (2014). Enamel wear caused by monolithic zirconia crowns after 6 months of clinical use. J Oral Rehabil.

[15] Mundhe K, Jain V, Pruthi G, Shah N (2015). Clinical study to evaluate the wear of natural enamel antagonist to zirconia and metal ceramic crowns. J Prosthet Dent.

[16] Shellis RP, Addy M (2014). The interactions between attrition, abrasion and erosion in tooth wear. Monogr Oral Sci.

[17] Chong BJ, Thangavel AK, Rolton SB, Guazzato M, Klineberg IJ (2015). Clinical and laboratory surface finishing procedures for zirconia on opposing human enamel wear: a laboratory study. J Mech Behav Biomed Mater.

[18] Choi BK, Han JS, Yang JH, Lee JB, Kim SH (2009). Shear bond strength of veneering porcelain to zirconia and metal cores. J Adv Prosthodont.

[19] Heintze SD (2006). How to qualify and validate wear simulation devices and methods. Dent Mater.

[20] Salazar MS, Pereira SM, Ccahuana VV, Passos SP, Vanderlei AD, Pavanelli CA (2007). Shear bond strength between metal alloy and a ceramic system, submitted to different thermocycling immersion times. Acta Odontol Latinoam.

[21] Abbasi M, Ebadian B, Aminianpour N (2023). Shear bond strength of colored and sandblasted zirconia to ceramic veneers fabricated by the pressing and layering techniques: an in vitro study. Int J Dent.

[22] Teng WS, Yew HZ, Jamadon NH, Qamaruz Zaman J, Meor Ahmad MI, Muchtar A (2024). Effect of porcelain veneering technique in bilayered zirconia on bond strength and residual stress distribution. J Mech Behav Biomed Mater.

[23] Sreekala L, Narayanan M, Eerali SM, Eerali SM, Varghese J, Zainaba Fathima AL (2015). Comparative evaluation of shear bond strengths of veneering porcelain to base metal alloy and zirconia substructures before and after aging - an in vitro study. J Int Soc Prev Community Dent.

[24] Nishigori A, Yoshida T, Bottino MC, Platt JA (2014). Influence of zirconia surface treatment on veneering porcelain shear bond strength after cyclic loading. J Prosthet Dent.

[25] Zandinejad A, Nasiry Khanlar L, Barmak AB, Ikeda M, Tagami J, Masri R. Shear bond strength of porcelain to milled and stereolithography additively manufactured zirconia with and without surface treatment: An in vitro study. J Prosthet Dent. 2023. 10.1016/j.prosdent.2023.02.007. 36932021

[26] Aboushelib MN, Kleverlaan CJ, Feilzer AJ (2008). Effect of zirconia type on its bond strength with different veneer ceramics. J Prosthodont.

[27] Vardhaman S, Borba M, Kaizer MR, Kim D, Zhang Y (2020). Wear behavior and microstructural characterization of translucent multilayer zirconia. Dent Mater.

[28] Steiner R, Scott S, Wiesmüller V, Lepperdinger U, Steinmassl O, Schnabl D (2024). Effect of zirconia surface conditioning before glazing on the wear of opposing enamel: an in vitro study. Clin Oral Investig.

[29] Ghaffari T, Hamedi Rad F, Goftari A, Pashazadeh F, Ataei K (2022). Natural teeth wear opposite to glazed and polished ceramic crowns: a systematic review. Dent Res J (Isfahan).

[30] Stawarczyk B, Özcan M, Schmutz F, Trottmann A, Roos M, Hämmerle CH (2013). Two-body wear of monolithic, veneered and glazed zirconia and their corresponding enamel antagonists. Acta Odontol Scand.

[31] Janyavula S, Lawson N, Cakir D, Beck P, Ramp LC, Burgess JO (2013). The wear of polished and glazed zirconia against enamel. J Prosthet Dent.

